# Clinical supervisors’ experiences and perceptions of the practice education facilitator role: a qualitative study

**DOI:** 10.1186/s12912-025-03293-5

**Published:** 2025-06-02

**Authors:** Cathrine Mathisen, Lena G. Heyn, Turid-Iren Jacobsen, Ida T. Bjørk, Elisabeth H. Hansen

**Affiliations:** 1 Faculty of Health and Social Sciences, Department of Nursing and Health Sciences, University of South-Eastern Norway, Drammen, Norway; 2 Faculty of Medicine, Institute of Health and Society, Department of Nursing Science, University of Oslo, Oslo, Norway

**Keywords:** Practice education facilitator, Clinical supervision, Supervisor support, Clinical learning environment (CLE)

## Abstract

**Background:**

In Norway, official bodies have emphasised the use of joint clinical-academic roles. One example is the practice education facilitator role, which promotes and supports the clinical learning environment by offering guidance to clinical supervisors and nursing students. Prior research indicates that such roles can enhance the clinical learning environment for nursing students, but recent research, particularly from the Norwegian context, is lacking.

**Aim:**

The study explored clinical supervisors’ experiences and views on how, under what circumstances, and why the role of practice education facilitators influences the clinical learning environment.

**Methods:**

A qualitative exploratory approach was chosen. Data were collected through semi-structured interviews and focus groups and analysed using thematic analysis. In total, 12 clinical supervisors from departments with an affiliated practice education facilitator were interviewed during the period from April to June 2021, from two larger acute care hospitals in Norway.

**Results:**

Thematic analysis resulted in three themes related to the participants’ experiences and views on how the practice education facilitator role influences the clinical learning environment: *Having someone to turn to*,* Benefitting from being an ‘insider*’, *and Champions of practice learning.*

**Conclusions:**

The results of this study show how practice education facilitators can enhance the clinical learning environment by offering support and guidance to clinical supervisors. A contextual factor found to influence the willingness of clinical supervisors to seek and receive support and guidance from these facilitators was the positioning of the facilitators as insiders within the clinical setting.

**Clinical trial number:**

Not applicable.

**Supplementary Information:**

The online version contains supplementary material available at 10.1186/s12912-025-03293-5.

## Introduction

To meet the requirements for a workforce possessing the competencies needed to provide safe and high-quality nursing care, nursing students need to develop a complex combination of knowledge, skills, and attitudes [1]. Opportunities to apply theoretical knowledge to authentic patient care are fundamental in this process [2, 3]. However, the clinical learning environment is complex and includes multiple interactive components [4] that influence the learning outcomes of nursing students during clinical placement [3].

In Norway, official bodies have called for increased use of joint roles between academic and healthcare organisations to strengthen the clinical learning environment and enhance the quality of nursing education (NOU, 2023). In this study, we use the term ‘practice education facilitator’ in a generic sense to refer to joint roles where postholders are employed by an academic institution, a healthcare provider, or both organisations to work within the clinical field to promote and support the clinical learning environment. Although prior research indicates that such roles can contribute to strengthening these environments [5], empirical knowledge of how these roles work within the Norwegian context is lacking. Given the emphasis on practice education facilitator roles in Norway, an understanding of how these roles influence the clinical learning environment of nursing students is needed to inform the further development of such roles. The current study is part of a larger research project focusing on the role of practice education facilitators.

## Background

Clinical training is recognised as a fundamental aspect of nursing education [6], but suboptimal learning environments in placements remain a concern [7]. A concept analysis conducted by Flott and Linden [4] identified four attributes of the clinical learning environment that may impact the learning experiences of nursing students in clinical practice: the physical space, psychosocial and interaction factors, organisational factors, and teaching and learning components. The physical space includes the layout of the clinical setting and the availability of equipment and resources. Psychosocial and interaction factors include the level of support and guidance provided by clinical staff, the level of anxiety and stress experienced by students, and interaction between nursing students, clinical supervisors, and staff. Organisational factors involve culture in relation to views on education and social and organisational policies, while teaching and learning components include factors such as the provision of relevant learning situations and nursing student engagement in learning [3, 4].

The quality of the clinical learning environment is known to have a significant impact on students’ learning and overall experience of clinical practice [3, 7]. A positive clinical learning environment promotes student engagement, motivation, and confidence and facilitates the development of critical thinking, problem-solving, and clinical reasoning skills [3]. Conversely, a negative clinical learning environment can lead to feelings of isolation, stress, and anxiety and may hinder students’ learning and development of clinical competence [7]. The clinical supervisor plays a crucial role in the clinical learning environment by providing support and guidance, teaching clinical nursing skills, and acting as a role model [8, 9]. In Norway, this role is usually performed by staff nurses, who supervise nursing students in addition to their clinical duties [10]. Along with a clinical supervisor, students also have a nurse educator appointed by the university who has overall responsibility for the student during placement. Typically, the nurse educator visits students in placement to talk about expectations the first week, as well as for midterm and final assessments. In addition, they often hold 1–3 group supervision meetings with students during placement. Assessment of students is usually performed collaboratively by the clinical supervisor and the nurse educator [11].

Given the crucial role of the clinical supervisor, circumstances such as high patient acuity, understaffing, and the demands of their own nursing duties, can serve as barriers for nursing student learning in clinical placements [6, 10]. Further, many clinical supervisors lack formal training for the role, which may influence the quality of both the supervision they provide and their assessment of students [12, 13]. Other challenges that have been reported include a lack of relevant learning situations due to a high degree of specialisation in healthcare services [10] and inadequate collaboration between academic institutions and placement providers [14]. The latter is often associated with a persistent challenge in nursing education known as the ‘theory–practice gap’ [14]. In a concept analysis conducted by Greenway et al. (p. 3) [14], the attributes of the theory–practice gap were identified as ‘relational problems between university and clinical practice’, ‘practice failing to reflect theory’, and ‘theory perceived as irrelevant to practice’. For nursing students, this may lead to discrepancies between what they learn at campus and what they experience in clinical practice [14]. These circumstances have led to concerns about nursing students’ development of clinical competence and their fitness for practice upon graduation [11, 15, 16].

An initiative that has been suggested to address the challenges in clinical practice and to reduce the theory–practice gap in nursing education is the use of practice education facilitators [17]. Much of the existing research focusing on practice education facilitators in contexts with education and placement models similar to those in Norway has been conducted in the UK and Ireland [5, 17–21]. The degree to which practice education facilitators are used in Norway is not known, but due to recommendations from official bodies in several reports since 2016 [22–24], there has been an increased emphasis on such roles.

In a previous study conducted as part of the same research project, we explored how practice education facilitators can contribute to strengthening the clinical learning environment from the perspective of those holding such roles. In this study we found that practice education facilitators are ideally placed to contribute to a stronger link between academic institutions and placement providers [25]. Similar findings have been found in several other studies conducted in UK and Ireland [18, 19, 26, 27]. In particular, clinical supervisors regard the support and guidance offered by practice education facilitators as a valuable resource [5, 28]. Challenges that have been reported include a lack of visibility and accessibility of the facilitators in the clinical area [5] and difficulties for postholders related to combining different cultures and ‘serving two masters’ [25, 29]. Other difficulties that have been identified are a lack of role clarity and insufficient preparation and support for the role, which can lead to a high burnout rate among practice education facilitators [30, 31].

Although practice education facilitators can be a valuable resource for clinical supervisors, there is a lack of recent research on their roles, and few empirical studies have explored these roles in the Norwegian context. Thus, this study explored clinical supervisors’ experiences and perceptions of how the practice education facilitator role influences the clinical learning environment for nursing students, under what circumstances, and why.

The research questions were as follows:

1. What are clinical supervisors’ experiences with the practice education facilitator role?
2. What are clinical supervisors’ views on how the practice education facilitator role can contribute to strengthening the clinical learning environment for nursing students?
3. What factors facilitate or hinder the benefits of using practice education facilitators in nursing education?

## Methods

### Design

We applied a qualitative approach with an exploratory design, as this open approach is appropriate for exploring participants’ experiences and perceptions [32]. Scientific realism as described by Pawson and Tilley [33], was used as a theoretical lens. A central concept in scientific realism is that an outcome produced by an intervention or social programme is not caused by the intervention itself but by individuals’ responses to the resources offered by the intervention. These responses are triggered by underlying mechanisms, which operates under certain contexts. This explains why an intervention can have different outcomes in different contexts [34].

Data were gathered through focus groups and in-depth individual interviews. Due to Covid-19, several focus groups were not carried out, and therefore, individual interviews were also performed. The principles of the consolidated criteria for reporting qualitative research (COREQ) were applied in this study [35].

### Participants and recruitment

A purposive sampling approach was used to recruit participants from two larger acute care hospitals in Norway. Inclusion criteria were staff nurses with experience as clinical supervisors for a minimum of one student and working in hospital wards with access to a practice education facilitator. Staff nurses without supervision experience and those who worked in wards without an affiliated PEF were excluded from the study.

Participants were recruited through nurse managers at the hospitals, who communicated information about the study to their staff nurses. The names and contact information of potential participants who were interested and had agreed to share their contact information were then provided to the researchers. None of the researchers had any prior personal or professional relationships with the participants. The first author sent a formal request and written information about the study by email directly to the potential participants to ensure that the managers were not directly involved in the recruitment process. A total of 26 nurses agreed to participate in the study. After several attempts to organise focus groups, 12 participants were able to participate. The participants had from 1 to 41 years of experience as nurses (mean 11.2 years), and all worked in different medical and surgical wards. Two participants had completed a 10-credit programme in clinical supervision, four participants had completed a short 2-day course, and seven participants had no formal training in supervision.

### Data collection

Data were collected from April to June 2021. We initially planned to collect data through focus groups only, but it proved to be difficult to conduct them as it was challenging to find suitable times for all participants due to high clinical workload. The research team therefore decided to conduct individual interviews with participants who had agreed to participate in the focus groups and still wished to contribute to the study even though the focus groups they had agreed to participate in were cancelled. Thus, data was collected through two focus groups and four individual interviews. The individual interviews and focus groups were conducted either face-to-face at their workplace or via digital communication platforms (Table 1). The first author conducted the individual interviews and moderated the two focus groups. The last author participated at the focus groups as an observer and took notes. A semi-structured interview guide was used to structure the interviews while leaving space for the participants to express their experiences and perceptions in their own words. The interview guide was developed for this study by the authors and covered the following areas: [1] experiences with the supervisor role [2], perceptions of the clinical learning environment [3], experiences with the practice education facilitator role [4], perceptions on how the practice education facilitator role can influence the clinical learning environment, and [5] experiences and perceptions of the factors influencing the benefits of the practice education facilitator role (see Supplementary file 1). The same interview guide was used for both the individual interviews and the focus groups. Follow up questions were asked to illuminate experiences and perceptions about how, where, when and why (or why not) the practice education facilitator role influenced the clinical learning environment. The interviews lasted between 59 and 75 min and were audiotaped.

**Table 1:** Data collection methods

Participant no.	Interview/platform
P1	Individual interview/Face-to-face
P2	Focus group 1/Teams
P3	“
P4	“
P5	Focus group 2/Face-to-face
P6	“
P7	“
P8	“
P9	“
P10	Individual interview/Zoom
P11	Individual interview/Zoom
P12	Individual interview/Zoom

### Data analysis

We employed reflexive thematic analysis following the six phases described by Braun and Clarke [36]: familiarisation; coding; generating initial themes; developing and reviewing themes; refining, defining, and naming themes; and writing the report. The first author transcribed the interviews verbatim, and the software programme NVivo (version 1.6) was used to manage and structure the text. Two of the authors (CM and EHH) read and reread the transcripts to become familiar with the data and wrote initial thoughts and ideas. In accordance with accepted practices for this approach [36], the first author coded the data by using inductive and semantic coding, while the others reviewed the process. Initial themes were generated by the first author and then reviewed and further developed by all authors. This was a process where we moved back and forth between the different phases. Finally, each theme was refined by generating clear definitions and suitable titles. Table 2 provides an example of the process.

**Table 2:** Example of the analysis process

Meaning unit	Code	Subtheme	Theme
The PEF at our unit, she has far more experience in the clinical field than the nurse educators. This is very useful when she participates in the assessment of students who are facing challenges in placement because her clinical experience enables her to understand what the challenge is in a certain way. [P8]	PEFs have a better ability to assess students than nurse educators because they are familiar with the clinical setting	PEF contribute to strengthened student assessment	Access to support and guidance
The PEF helped us in a situation when I had a student who struggled with the language. This was a delicate situation, and it was very useful to have someone outside the ward to call, that could come and help us solve this situation in a proper way. [P11]	PEFs are useful when challenges occur in the supervisory relationship	PEFs offer support and guidance in challenging student situations	
I have participated in a few of these meetings that the PEF organiser for the clinical supervisors. And one of the PEFs also followed me at the ward and provided supervision to me as a supervisor when I supervised the student. I believe this kind of support helps us to become better supervisors [P12]	The PEF offers support and guidance for clinical supervisors in their role	Clinical supervisors are supported in their role	

*PEF = Practice Education Facilitator

### Ethical considerations

This study was conducted in accordance with the Declaration of Helsinki, and ethical approval was obtained from SIKT, formerly known as the Norwegian Centre for Research Data, project number 255 − 238. The participants received both written and oral information about the study, and informed written consent was secured from all participants. Ahead of the interviews, the participants were informed that they could withdraw from participation at any time, without any negative consequences. To ensure confidentiality for all participants, names, gender, workplaces, or other identifying information was not included in the transcripts.

## Results

The key findings generated from the thematic analysis resulted in three themes related to the participants’ experiences and views on how the practice education facilitator role influences the clinical learning environment: Access to support and guidance; Benefitting from being an ‘insider’; and Champions of practice learning. The following descriptions of the themes include quotations from the interviews to illuminate the participants’ voices.

### Access to support and guidance

This theme reflects different ways in which the participants experienced that the practice education facilitator role provided them access to support and guidance, which in turn helped them support students in placement.

Although nurse educators hold the primary responsibility for students in placement, the participants conveyed that the role of the clinical supervisor carries a considerable responsibility, particularly in relation to student assessment. This is because nurse educators, who are not present in the clinical field, must rely on the observations of clinical supervisors for their assessments. Therefore, the participants valued the introduction of the practice education facilitator role, as it provided a second opinion when they were uncertain or dealing with students who had struggled in placement. However, the benefits of the resource provided by the practice education facilitator were identified to be contingent on the postholder’s familiarity with the clinical setting. Practice education facilitators who were experienced clinicians and familiar with the specific clinical setting were perceived as ‘insiders’, able to grasp the situation and identify challenges in the supervision in a way that nurse educators, as ‘outsiders’, could not. When asked to assist in challenging student situations, practice education facilitators would often make their own individual assessment of the student. This was perceived by the participants as reassuring, both for themselves and for the students.

In situations like this, when there are challenges, the opportunity to involve the practice education facilitator is very ensuring, four eyes see more than two, that’s how it is. (P1, individual interview)

When clinical workloads were high, clinical supervisors were able to reach out to the practice education facilitator when they found it difficult to provide the necessary support and guidance to students. In such situations, some practice education facilitators would offer to assist students in caring for a patient. This was beneficial for the clinical supervisor, who could then focus solely on their clinical duties for a while, creating a win-win situation for both students and their supervisors. The supervisors’ burden was eased, and the practice education facilitator ensured that students’ learning wasn’t compromised during such times. The practice education facilitator also served as a go-to person for clinical supervisors when a student needed a more experienced supervisor or when they required help in providing relevant learning situations for students.

There are limited learning opportunities at an outpatient surgical ward, so I contacted the practice education facilitator because my student wanted to catheterise a patient. (P1, individual interview)

The participants appreciated the practice education facilitator role, as it gave them access to support and guidance from someone with dedicated responsibility for practice learning within the clinical setting. Having access to this resource helped them feel more confident in the supervisor role, and for some, it even enhanced their motivation to take on responsibilities as a clinical supervisor.

It’s very useful to have the opportunity to just call the practice education facilitator and discuss the student. How do you experience the student? Is this stupid, or do you have the same impression? (P3, focus group)

Access to support and guidance in challenging supervision situations did more than help this participant manage these situations appropriately. It also created valuable learning opportunities that contributed to their development as a supervisor. This access to support therefore contributes to both immediate problem-solving assistance and professional growth.

### Benefitting from being an ‘insider’

This theme elucidates how the practice education facilitator’s ‘insider’ status, stemming from their familiarity with and presence in the clinical area, creates opportunities for action and influence that are not available for a nurse educator with an ‘outsider’ status.

Before the introduction of practice education facilitators, the participants indicated they felt a lack of formal support in their supervisor role. Even though a designated nurse educator was available for them to contact in case of challenges, they perceived this role to be more formal. As a result, they were hesitant to reach out for what they described as ‘minor things’.

We can call them [the nurse educator] as well, but that’s something we do when there is something serious going on. We can’t call them to ask for help supervising students in clinical skills. (P11, individual interview)

This reluctance to reach out to the nurse educator was reinforced by the geographical distance between the university and the clinical site, as well as the perceived heavy workload at the university.

Well, if, I’m going to schedule a meeting with the nurse educator, it becomes something like ‘Yes, I’m available next Thursday’. By then, half of the placement period has passed before we manage to address the problem. (P8, focus group)

In contrast to nurse educators, practice education facilitators were more accessible because they were located in the same place and were employees of the healthcare institution. The participants found it easier to reach out to someone they knew, especially for ‘minor’ issues or when they needed immediate guidance or support. Their willingness to engage with the practice education facilitator was significantly influenced by their personal relationship with and knowledge of the individual in that role. This highlighted the importance of a practice education facilitator’s visibility in the clinical setting. Without a clear understanding of the role and its potential benefits, as well as a personal connection with the individual holding the role, the participants were less likely to seek out the support provided by the practice education facilitator. Therefore, the participants stressed the need for facilitators to actively promote their role and clarify the support they could offer to clinical supervisors.

I didn’t realise what the practice education facilitator could offer before this meeting [focus group], and I believe that’s why no one at my ward has used the opportunity to seek guidance and support from this person. Now, when I’ve heard your experiences and understand what they can offer, I will for sure call our practice education facilitator the next time I face a challenging student situation. (P9, focus group)

### Champions of practice learning

This theme describes how the practice education facilitators’ dual familiarity with both the clinical area and education enabled them to promote practice learning in the clinical field.

Now, when we have more focus on practice learning, we’re more aware. It’s not so much ‘… Ugh, are we getting students again …’. We have a more positive attitude toward clinical supervision. (P6, focus group)

The participants found the academic formulations in the university’s written material challenging to understand. However, the practice education facilitators, equipped with their understanding of the education, were able to translate this content into more accessible language for clinical supervisors. This translation, facilitated by the practice education facilitators’ dual familiarity with both clinical and academic aspects, helped the participants develop a deeper understanding of the assessment tool.

Participants perceived practice education facilitators as having a deeper understanding of their expectations and struggles compared to nurse educators, thanks to their familiarity with the clinical setting. This understanding enabled a rapport, making collaboration with practice education facilitators easier for the participants than with nurse educators.

When I talk to them [a practice education facilitator] I feel understood in a different way. Well, I do believe that nurse educators also understand that we’re busy and that it’s difficult to juggle patient care and supervising students. But the dialogue is not the same, it’s difficult to put a finger on what it is. (P9, focus group)

Thus, the unique access that practice education facilitators have to clinical supervisors and the clinical field, thanks to their position as insiders, empowers them to champion practice learning effectively in the placement setting.

The practice education facilitators who are working here are excellent clinicians and closely connected with the education. They’re able to come up with relevant arguments and able to influence the organisation in a different way than a nurse educator who is not working as a clinician. (P12, individual interview)

Familiarity with organisational structures and knowing people were perceived as crucial for practice education facilitators. Additionally, possessing good interpersonal skills, flexibility, and a solution-oriented approach were essential for them to establish a role within the organisation that enabled them to achieve the desired results. Thus, the benefits of the role were believed to be heavily dependent on the person who had the job.

## Discussion

This study sought to provide insights on how the practice education facilitator role influences the clinical learning environment for nursing students, under what circumstances, and why by exploring clinical supervisors’ experiences and perceptions of the role. The findings showed that the practice education facilitator role was viewed as a valuable resource by clinal supervisors. Especially in relation to student assessment, the participants appreciated having someone in the clinical field to turn to for support and guidance. This finding highlights a well-known challenge in nursing education related to clinical practice occurring in a different setting than the theoretical education [12]. Although assessment is a joint responsibility of the nurse educator and clinical supervisor, nurse educators often have limited time to spend in the clinical setting [37]. Thus, the assessment process is often highly dependent on clinical supervisors’ judgement, which is concerning considering prior research implying that they are inadequately prepared for the supervisor role [3, 7].

A challenge reported by researchers is that clinicians may encounter difficulty in understanding written material provided by the university. For instance, Christiansen et al. [37] found that it was challenging for clinical supervisors to tie the learning outcomes described in an assessment form to clinical situations because the learning outcome formulations were general and lacked concrete guidelines. Academic language may be unfamiliar to clinical supervisors and increase the gap between theory and practice. In this study, practice education facilitators were able to guide clinical supervisors in how to use the assessment form by showing them how general learning outcomes could be tied to typical clinical situations in their context.

An interesting finding in this study was that clinical supervisors were reluctant to contact nurse educators when they faced challenges in the supervisory process, unless there were major concerns. The reasons appeared to be multifaceted, but distance, both geographically and figuratively between the academic institution and the placement site, was an issue. This is worrying, considering that ‘failing to fail’ is a well-known and persistent concern in nursing education [38]. In a study by Hauge et al. [11] in the Norwegian context, 16.8% of the clinical supervisors reported that they had passed a failing student. Similar numbers have been reported in international studies [39]. Hence, passing nursing students who do not meet requirements is a real problem in nursing education, one which ultimately presents a threat to patient safety [38]. Reasons for ‘failing to fail’ have been identified include lack of support from the nurse educator in the assessment process and difficulties in prioritising supervision for clinical supervisors due to heavy clinical workloads. This in turn causes clinical supervisors to give the student the benefit of the doubt [11, 38]. Thus, the use of practice education facilitators, who are present in the clinical area and able to develop rapport with clinical supervisors in a way that nurse educators may not be able to, could ultimately contribute to patient safety.

A key argument for using practice education facilitators in nursing education is that it leads to strengthened partnership and collaboration between the university and the placement provider [17, 40]. The findings of this study showed that practice education facilitators, by virtue of their insider position, provided a valuable link between education and the clinical area, thus creating a unique platform for action. This aligns with Jowett and McMullan [41], who also found that this insider status enables practice education facilitators to provide this vital link. Moreover, a study by Hancock et al. [26], found that practice education facilitators perceived themselves as ideally positioned to act as liaisons between the two settings because they have a ‘foot in each camp’, which underscores the importance of their insider status. This is interesting given the challenges in creating coherence between classroom-based and practice-based learning for nursing students, which have been reported and linked to the so-called theory–practice gap [14, 42]. The lack of clinical credibility of nurse educators has been highlighted as a barrier due to a perception among clinicians that they do not really know what they are talking about because they are not doing it themselves [43]. This study confirms that having ‘clinical credibility’ is an asset for practice education facilitators which may put them in a position to reduce the gap between theory and practice.

Even though the practice education facilitator role was found to be a valuable resource for clinical supervisors, the findings also revealed barriers that need to be addressed for the full potential of the role to be realised. For instance, the resources offered by the practice education facilitator were not known in all areas, and thus, not all clinical supervisors had used the opportunity to seek guidance and support. The reasons for this were not identified in this study, but others have pointed to a lack of resources, as practice educators have too large an area to cover and not enough hours dedicated for the role [5, 26]. Another potential barrier that was identified in this study was the fact that the benefits of the role were contingent on the person who had the job. As mentioned earlier, being able to develop rapport with clinicians was crucial, and a lack of certain attributes such as clinical credibility might diminish the benefits of the role.

### Limitations and strengths

The current study provides valuable insights into how the practice education facilitator role can strengthen clinical learning environments. However, one limitation is the low number of participating institutions, which, along with the common difficulty in generalising qualitative research data, affects the study´s overall applicability. Despite this, it was a strength that the participants came from many different departments, thereby representing a variety of settings.

Our original plan was to collect data solely from focus groups, but the combination of individual interviews and focus groups proved to be a strength of this study. The individual interviews provided opportunities to explore the phenomenon of interest in more depth, while the discussions in the focus groups offered valuable insights that would not have emerged without the group interaction.

## Conclusion

Findings in this study indicate that the practice education facilitator role has the potential to enhance educational quality by assisting clinical supervisors in their role. Clinical supervisors need support and guidance, but the participants in this study were more likely to seek and receive support and guidance from someone they knew and perceived to have clinical credibility. Thus, the practice education facilitators’ unique position as insiders in the clinical area provided them with opportunities that those with an outsider position would not have. The insider position also provided the facilitators opportunities to strengthen the clinical learning environment by acting as champions for practice learning and improving coherence between theory and practice. However, the benefits of the role were dependent on the person who had the job, and thus, when recruiting nurses to such positions, it is important to be aware of the importance of certain personal attributes required to succeed in the role.

## Electronic supplementary material

Below is the link to the electronic supplementary material.

Supplementary Material 1

Supplementary Material 2

## Data Availability

The data from the audio-recorded interviews/focus groups are available from the corresponding author upon reasonable request.
