# Supplementary material for: Clinical supervisors’ experiences and perceptions of the practice education facilitator role: a qualitative study
Source: BMC Nurs. 2025 Jun 2;24:626. doi: 10.1186/s12912-025-03293-5 (PMC12128264; doi:10.1186/s12912-025-03293-5)
Supplement: Supplementary file 2 — Supplementary Material 2 [file 12912_2025_3293_MOESM2_ESM.docx]

**Supplementary file 1. Semi-structured interview guide**

1. **Introduction: Setting the Scene**

- Introduction of the researchers
- Background information about the research project
- Explanation of the purpose of the study
- Confidentiality and anonymity
- Clarification that participation is voluntary

1. **Background information**

(Recording has started)

- Age (20-29, 30-39, 40-49, 50-60, over 60)
- Years of experience as a nurse
- What type of department/ward do you work in?
- How many years have you worked in this department?
- Have you been supervisors before? Approximately how many times?
- Have you attended courses related to the supervisor role?

1. **Main interview questions**

**Participants experiences with the supervisor role**

- Could you briefly describe your experiences of being a clinical supervisor for nursing students?

**Perceptions of the clinical learning environment (CLE)**

- What do you think are the most important things nursing students should learn during clinical placement?

Follow-up: Can you provide examples or situations where these skills and knowledge are particularly crucial?

- What is needed for students to achieve this?

Follow-up: What resources, support, or conditions are essential for facilitating these learning outcomes?

- How do you try to facilitate students in gaining this knowledge or these skills?

Follow-up questions to explore contextual conditions influencing the supervision of students

**Experiences with the Practice Education Facilitator (PEF) role**

*Introduction:* *The PEF role has been established at your hospital to act as a link between education and the clinical field, and they also have overarching responsibility for clinical placements at the hospital and for offering provision of support and guidance to clinical supervisors and nursing students.*

- Are you familiar with this role?
- Can you describe your experience with the PEF role?
- Have you used any of the resources provided by the PEF?

Follow-up: in what situations have they been in contact with the PEF, why/why not, their experiences with the resources provided by the PEF

Follow-up: Management of difficult or challenging student situations

- - What significance did the guidance and support offered have in this situation? (for the student, for the supervisor)
  - Did you involve the nurse educator? Why/why not?

**Experiences and perceptions of the factors influencing the benefits of the practice education facilitator role**

*Introduction: As I mentioned, the objective of this study is to explore the PEF role and how this role may contribute to strengthen the clinical learning environment for nursing students, and if so – how, under what circumstances and why (or why not)*

- What factors do you think are important for such roles to be a resource in the CLE?
- Are there any factors you think could negatively impact the usefulness of such positions?
- Do you have any suggestions or recommendations for the further development of such roles?

1. **Summary**

To conclude, I would like to summarise the key points that were discussed in this interview.

- Did I understand you correctly? Do you have anything to add?
- Do you have any final thought you would like to share about the use of PEFs in nursing education?
- Thank the participants for their participation
- STOP RECORDING
